# Cell metabolism-based optimization strategy of CAR-T cell function in cancer therapy

**DOI:** 10.3389/fimmu.2023.1186383

**Published:** 2023-06-05

**Authors:** Wenshuai Li, Xuanxuan Pan, Lirong Chen, Haoshu Cui, Shaocong Mo, Yida Pan, Yuru Shen, Menglin Shi, Jianlin Wu, Feifei Luo, Jie Liu, Na Li

**Affiliations:** ^1^ State Key Laboratory of Quality Research in Chinese Medicine, Macau University of Science and Technology, Macao, Macao SAR, China; ^2^ Department of Digestive Diseases, Huashan Hospital, Fudan University, Shanghai, China

**Keywords:** cell metabolism, cancer therapy, optimization strategy, CAR (chimeric antigen receptor) T cells, immunity therapy

## Abstract

Adoptive cell therapy (ACT) using chimeric antigen receptor (CAR)-modified T cells has revolutionized the field of immune-oncology, showing remarkable efficacy against hematological malignancies. However, its success in solid tumors is limited by factors such as easy recurrence and poor efficacy. The effector function and persistence of CAR-T cells are critical to the success of therapy and are modulated by metabolic and nutrient-sensing mechanisms. Moreover, the immunosuppressive tumor microenvironment (TME), characterized by acidity, hypoxia, nutrient depletion, and metabolite accumulation caused by the high metabolic demands of tumor cells, can lead to T cell “exhaustion” and compromise the efficacy of CAR-T cells. In this review, we outline the metabolic characteristics of T cells at different stages of differentiation and summarize how these metabolic programs may be disrupted in the TME. We also discuss potential metabolic approaches to improve the efficacy and persistence of CAR-T cells, providing a new strategy for the clinical application of CAR-T cell therapy.

## 1 Introduction

T cells are the key players in adaptive immunity. Having matured within the thymus, lymphocytes enter the circulation as naïve T cells. Upon T cell antigen receptor (TCR)-mediated recognition of antigens, along with costimulatory signals and cytokines, naïve T cells become activated, rapidly proliferate and differentiate into effector cells. After pathogen clearance, most effector cells undergo activation-induced cell death, leaving behind a small pool of long-lived, quiescent memory T cells that respond to subsequent antigen challenges with enhanced reactivity (1). However, during persistent stimulation, such as chronic infections, autoimmunity, and cancer, T cells may become exhausted, characterized by the loss of effector functions, elevated and sustained expression of inhibitory receptors (IRs), and altered epigenetic and transcriptional profiles (2). Each of these phases is characterized by unique metabolic demands for energy, macromolecule biosynthesis and redox, accompanied by dynamic changes in cellular metabolism, which is also known as metabolic reprogramming (3). Moreover, T cell metabolism is increasingly being recognized as a driver of quiescence, activation, or exhaustion, rather than merely following changes in these states. Manipulating nutrients and energy available to the cells can affect and change T cell fate decisions and effector functions (4).

The application of chimeric antigen receptor (CAR) T cells in cancer therapy is an emerging immunotherapy approach that has gained increasing attention. CAR-T cells are genetically engineered to express a single-chain variable fragment (scFv) derived from the variable domains of antibodies, combined with transmembrane and intracellular signaling domains, that specifically recognize tumor antigens in a non-MHC-restricted manner (5). To date, CAR-T cells have demonstrated great success in eliminating hematological malignancies. However, CAR-T cell therapy for solid tumors still has limitations and deficiencies, and the reason for these shortcomings is partly due to the metabolic stress imposed by the tumor’s hostile microenvironment. Furthermore, insufficient tumor vascularization, as a result of disorganized blood vessel networks, contributes to the depletion of nutrients, hypoxia, acidosis, and the accumulation of metabolic waste within the tumor microenvironment (TME) (6, 7). Moreover, the increased nutrient and oxygen demands of tumor cells further augment the deterioration in metabolism that may inevitably impair T cell fitness and effector functions, allowing tumor progression (8, 9). Therefore, it is of great significance to identify the key metabolic targets that help overcome these hurdles and improve CAR-T cells fitness in the TME of solid tumors. In this review, we introduce the metabolic requirements at different stages of T cells differentiation, summarize the complicated effects of metabolic disorders in the TME on T cells and discuss how metabolic interventions might be exploited to enhance the CAR-T cells antitumor response to provide new insights into improving the therapeutic efficacy of CAR-T cell therapy in solid tumors.

## 2 Metabolic characteristics of T cells in different differentiation stages

The differentiation status of T cells is significantly related to the therapeutic effect and persistence of CAR-T cells. The different stages of T cells differentiation include Naïve T cells (T_N_), stem cell central memory T cells (T_SCM_), central memory T cells (T_CM_), effector memory T cells, effector T cells and terminal differentiation T cells. Because T cell subsets with low differentiation (such as T_N_, T_CM_ and T_SCM_) have strong self-renewal ability, CAR-T cells products with high contents of T_N_, T_CM_ and T_SCM_ cells show excellent antitumor responses and persistence *in vivo (* 10, 11). The differentiation status of T cells is closely related to the metabolic phenotype, so it is important to understand how metabolism shapes T cell differentiation.

### 2.1 Naïve T cells (Tn)

Naïve T cells(T_n_)remain in a quiescent state, with low metabolic demands, and use predominantly mitochondrial oxidative phosphorylation (OXPHOS) fueled by small amounts of glucose, amino acids and fatty acids to generate ATP (12, 13). The quiescent metabolic status of naïve T cells is not a default setting but is actively maintained, requiring both extrinsic signals *via* tonic TCR (14), IL-7R (15) and sphingosine 1-phosphate 1 receptor (S1P1R) (16) to preserve Glut1 (glucose transporter 1) expression and increase glucose and amino acid catabolism while suppressing mitophagy to maintain their mitochondrial content and OXPHOS, as well as intrinsic low activity of the PI3K/Akt/mammalian target of rapamycin (mTOR) signaling pathway (17, 18). These pathways are often targeted therapeutically to inhibit autoreactive T cells, as in the case of autoimmune and allergic disease. It is worth mentioning, however, that an *in vitro* study suggested that naïve T cells from patients with metastatic melanoma exhibit increased transgene expression and proliferation (15), and translational data from CAR-T cell therapy trials indicate that the presence of naïve T cells in the premanufactured product is important to clinical response and persistence (19). Moreover, CAR-T cells generated by a homogenous naïve T-cell phenotype exhibit increased antitumor potential (20).

### 2.2 Effector T cells (Teff/Treg)

Upon activation, T cells rapidly engage in multiple metabolic pathways to support their growth, proliferation, and effector function. These pathways include aerobic glycolysis, pentose phosphate pathway (PPP), glutaminolysis and the hexosamine biosynthesis pathway (HBP), as well as mitochondrial biogenesis that facilitates oxidative phosphorylation (OXPHOS) and one-carbon metabolism.

T cell activation is accompanied by an increase in glycolytic flux *via* the enhanced expression of GLUT1 and certain metabolic enzymes (such as hexokinase2, HK2; phosphofructokinase1 PFK1; pyruvate kinases isoenzyme M2, PKM2; and lactate dehydrogenase A, LDHA) following TCR and costimulatory signals in a mTORC1-dependent manner (21). In contrast to T_N_, which breaks down glucose to fuel TCA and OXPHOS to maintain basal energetic needs, increased glucose uptake in Teff is mostly converted to lactate or allocated to the proximal branch pathway to provide building blocks for synthesizing intermediates or macromolecules, although at the expense of low net ATP gain (3). The shunting of glucose into the PPP pathway provides ribose-5-phosphate (R5P) for ribonucleotide synthesis and NADPH production. NADPH is critical not only for the biosynthesis of fatty acids and cholesterol but also for the regeneration of the antioxidant glutathione and acts as a substrate for NADPH oxidase, so it plays an important role in the maintenance of a favorable cellular redox environment (10). In addition, glucose-derived pyruvate transforms into acetyl-CoA, promoting the synthesis of lipids and cholesterol through the mevalonate pathway. ACC1, an enzyme that catalyzes the conversion of acetyl-CoA to malonyl-CoA and subsequent long-chain FA synthesis mediated by fatty acid synthase (FASN), appears dispensable for acquiring CD8+ T cells effector function but plays an indispensable role in their accumulation by influencing the survival of proliferating cells (22). Additionally, the relationship between glucose metabolism and autophagy has been initially explored, and knocking out autophagy-related genes, such as Atg5, can promote glycolysis in CD8+ T cells (23).

Induction of cytokine expression and effector function in Teffs is also a glucose-dependent process. The glycolytic metabolite phosphoenolpyruvate (PEP) is critical in sustaining T cell receptor-mediated Ca^2+^-NFAT signaling by repressing sarco/ER Ca^2+^-ATPase (SERCA) activity. Thus, overexpression of phosphoenolpyruvate carboxykinase 1 (PCK1) can increase PEP production, resulting in bolstered effector functions and antitumor responses. The glycolysis enzymes GAPDH (24) and LDHA (25) impact the expression of IFN-γ through posttranscriptional and epigenetic mechanisms. Furthermore, glucose and glutamine metabolism through the hexosamine biosynthesis pathway (HBP) increases the production of the amino sugar uridine diphosphate N-acetylglucosamine (UDP-GlucNAc) for posttranslational modification. For instance, in activated T cells, O-GlcNAcylation of the NF-kB subunit is necessary for its DNA binding activity and expression of cytokines (26).

Glutaminolysis generates 2-ketoglutarate (2-KG), a crucial intermediate for TCA cycle anaplerosis to fuel mitochondrial OXPHOS, and glutathione (GSH), which neutralizes reactive oxygen species (ROS) and is essential for NFAT nuclear localization and T cell proliferation. In addition to glutamine, other amino acids play an indispensable role in Teffs as well, which has been comprehensively reviewed recently (27, 28).

Contrary to conventional effector T cells, an *in vitro*-induced regulatory T cell fate is favored when oxidative phosphorylation (OXPHOS) and fatty acid oxidation (FAO) pathways are preferentially engaged in glycolysis (29). However, Treg cells that develop *in vivo* exhibit a similar reliance on glycolysis-driven lipogenesis for proliferation and function as Teff cells (30). In contrast, human Treg cells isolated *ex vivo* display a highly glycolytic phenotype and engage in both glycolysis and FAO when cultured *in vitro* (31). These differences may be attributed to specific developmental programming and/or context-dependent external cues.

### 2.3 Memory T cells (Tm)

Four distinct subsets of memory T cells have been characterized based on their phenotype, migration pattern, and functional properties: central memory cells (Tcm), effector memory T cells (Tem), tissue-resident memory T cells (Trm), and stem cell-like memory T-cells (Tscm). Previous studies have established that less differentiated Tcm and Tscm subsets exhibit better expansion, survival, persistence, and, therefore, heightened antitumor activity in CAR-T immunotherapy. Understanding the specific characteristics and behaviors of these subsets is crucial for optimizing CAR-T cell therapy and improving clinical outcomes.

#### 2.3.1 Tcm

Tcm cells are a subset of memory T cells that promote migration into lymph nodes (LNs) and quickly proliferate when they are re-exposed to antigens, producing TNF-α and IL-2 and expressing CD45RO, CD62L, CD28, CD27, CCR7, CD127, CD11a, IL-18Rα, CXCR4, and CXCR3 but not CD45RA (32). An increased frequency of Tcm cells is an important correlate of antitumor T cell responses following ACT. Therefore, numerous studies have emphasized the importance of modifying current protocols for T cell activation and expansion to preserve or induce a long-lived memory T cell signature while ensuring sufficient expansion (33).

Metabolically, Tcm cells switch from glycolysis back to FAO and OXPHOS, which is regulated by AMP-activated kinase (AMPK) signaling, and their long-term survival is promoted by an increased mitochondrial spare respiratory capacity (SRC). SRC refers to the reserve energy-generating capacity available in the mitochondria beyond the basal state and is essential for longevity and optimal T cell function. Peroxisome proliferator-activated receptor gamma coactivator 1-alpha (PGC-1α) is a key regulator of mitochondrial biogenesis and modulates OXPHOS and FAO by inducing the expression of nuclear respiration factor 1 (Nrf-1) and mitochondrial transcription factor A (Tfam) (34). Enforced PGC-1α expression promotes Tcm formation, and adoptive transfer of CD8+ T cells overexpressing PGC-1α leads to an improved antitumor response in melanoma-bearing mice (35). In addition, Tcm cells demonstrated a significantly larger mitochondrial mass and SRC than Teff and Tn cells. IL-15, another critical cytokine that regulates Tm cell homeostasis and self-renewal, enhances SRC by promoting mitochondrial biogenesis and the expression of CPT1a.

Furthermore, another cycle has been elucidated recently: the gluconeogenesis–glycogenolysis cycle, which activated by cytosolic phosphoenolpyruvate carboxykinase (PckCK1, the enzyme converting oxaloacetate (OAA) to phosphoenolpyruvate (PEP) and is highly expressed in Tcm) to fuel flux into the PPP, leading to enhanced generation of NADPH, thus protecting against ROS to support the long-term survival of CD8+Tm cells (36). Mechanistically, mitochondrial acetyl-CoA is diverted to the ketogenesis pathway in Tm cells, generating β-hydroxybutyrate (BHB), which epigenetically modifies Lys K9 of histone H3 (H3K9) of Foxo1 and Ppargc1a (which encodes PGC-1α) with β-hydroxybutyrylation, resulting in upregulation of PckCK1 expression (37). Translational studies have confirmed that BHB enhanced OT-I T-cell-mediated destruction of OVA-B16 melanoma and prolonged mouse survival both in systemic injection and in an ACT model (37).

#### 2.3.2 Tem

Tem cells are commonly found in nonlymphoid tissues and continuously circulate through the blood, expressing CD45RO, CD122, CD95, KLRG1, LFA-1, IL-18Rα, and chemokine receptors but are negative for CD62L, CCR7, and CD31. These cells are considered committed progenitor cells that undergo terminal differentiation after a limited number of divisions.

Compared to Tcm cells, Tem cells use OXPHOS in conjunction with glycolysis to generate energy, showing a higher extracellular acidification rate (ECAR), a marker of lactic acid production and glycolysis, and maintaining significantly less mitochondrial mass with lower SRC and a reduced basal rate of cellular respiration (38, 39). The abundance of key glycolytic enzymes, including HK1, GAPDH, PKM2 and LDHA, was found to be similar in Tn and Tem cells. However, GAPDH activity was greater in Tem cells than in their naïve counterparts (39). Persistent elevated glycolytic metabolism due to von Hippel-Lindau (Vhl) deficiency could support the development of a persistent Tem cells population, which induced constitutive hypoxia-inducible factor (HIF) activation (38). In Tem cells, glycolysis plays a major role in providing carbon for generating citrate in the mitochondria, which ultimately drives the histone acetylation necessary for IFN-γ production (40). The rapid and stable activation-induced glycolysis of CD8+ Tem cells depends on mTORC2-AKT signaling but not mTORC1, which primarily controls the metabolism of activated effector cells (41).

#### 2.3.3 Tscm

Tscm cells are a long-lived human memory T-cell population that displays enhanced self-renewal and multipotent capacity to derive Tcm, Tem and Teff cells, which exhibit a Tn-like phenotype but coexpress stem-cell markers and high levels of anti-apoptotic molecules (42). Moreover, there is growing evidence suggesting that adoptively transferred Tscm cells have enhanced antitumor activity and are more therapeutically effective than conventional Tcm and Tem cells in both preclinical models and clinical trials (43–45).

Tscm cells prefer OXPHOS to glycolysis over all other T cell subsets and possess higher basal and maximal respiration capacity, increased mitochondrial amounts, PGC-1αA expression, ATP content, and the NADH/NAD ratio (46). In addition, Tscm cells contain larger amounts of intracellular neutral lipid droplets, which can be utilized by FAO to fuel OXPHOS upon antigen stimulation. Tn cells and, to a lesser extent, Tscm cells retain a substantial reservoir of GSH to maintain a favorable redox state (47). Buffering ROS with the antioxidant N-acetylcysteine (NAC) during Tn cell activation prohibits terminal differentiation while allowing expansion and generation of Tscm cells, which establish long-term memory *in vivo* and exert more potent antitumor immunity in a xenogeneic model with CD19-specific CAR (47). However, excessive ROS scavenging by NAC may restrain T cell activation and proliferation, and tightly controlled ROS levels are needed.

#### 2.3.4 Trm

Recently, CD69+CD103+ Trm cells were identified as nonrecirculating immune cells that reside in peripheral tissues for local immune surveillance, especially in epithelial barrier tissues such as the skin, lung and gut (48). In contrast to Tcm cells, Trm cells generated from viral-infected skin depend upon exogenous fatty acid uptake and subsequent oxidative metabolism *via* cell fatty acid-binding protein 4 (FABP4) and FABP5 for their survival and long-term persistence. Trm cells even have a higher uptake of FFAs than actively proliferative Teff cells, and PPARγ is an upstream regulator of Fabp4 and Fabp5 gene expression (49). Moreover, the stress-responsive transcription factor Bhlhe40 is critical for Trm cells survival and production of effector molecules (IFN-g, GzmB, and TNF) by maintaining the mitochondrial fitness and metabolism of Trm cells, thereby promoting an active chromatin state for their residency and function (50). Of note, Trm cells have been identified as members of CD8+ tumor infiltrating lymphocytes (TILs) in patients with various solid tumors, and a Trm cell gene signature has been associated with a favorable prognosis and an increased likelihood of a response to anti-PD1 therapy in lung, breast, and melanoma cancers (48). Boosting Trm cells formation and metabolic fitness might be an attractive strategy to improve CAR-T cell therapy, for which a better understanding of the identity and role of the tissue-specific factors required for the generation and maintenance of activated Trm cells is needed.

## 3 T cells shaped by the metabolically challenged TME

Solid tumors typically develop a hostile microenvironment characterized by an irregular vascular network, resulting in a correspondingly poor oxygen and nutrient supply as well as a low capacity for metabolite removal. Prodigious appetite of tumor cells for nutrients and generation of toxic metabolites further deteriorates this situation, resulting in a metabolically challenged TME. Altogether, hypoxia, nutrient depletion, metabolite accumulation and redox imbalance in the TME can lead to T cells hypo-responsiveness and tumor progression, highlighting the critical role of appropriate metabolic remodeling in these cells. Furthermore, metabolism plays a critical role in driving T cell survival, activation, development, proliferation, differentiation, and antitumor effector function.

### 3.1 Glucose

Metabolic competition between immune cells and tumor cells is well known, with the Warburg effect being a typical example. Glycolysis in tumor cells as well as the downregulation of GLUT1 can limit T cell metabolism (51), which results in lower expression of mTOR, reduced expression of glycolysis-related genes, and decreased IFN-γ production, all of which contribute to the impairment of T cell’s antitumor abilities. Additionally, the glucose-deficient environment downregulates MHC-I expression on tumor cells, resulting in diminished T cell function because of the insensitivity to IFN-γ and the IFN-γ-STAT1 pathway, which can be rescued by a PIK3 inhibitor (52). For T cells themselves, glucose deprivation reduces the intracellular concentration of phosphoenolpyruvate (PEP), and the reduction in PEP is not only due to the lack of glucose but also due to the downregulation of enolase 1 (53). PEP deficiency restores the activity of sarco/ER Ca^2+^-ATPase (SERCA), inducing the endoplasmic reticulum to take up Ca ions, which inhibits the Ca^2+^/NFAT1 pathway (NFAT1 is a nuclear factor of activated T cells) in a glucose restriction environment (54). Acetate can enhance histone acetylation and chromatin accessibility when transformed to acetyl-CoA, thus promoting the production of IFN-γ (55) and leading to an increase in the antitumor activity of CD8+ T cells, which can be applied to CAR-T cell therapy.

Tumor cell glycolysis also induces high expression of PD-1 in T cells, inhibiting the efficacy of immune checkpoint therapy (56). The high expression of PD-L1 in tumor cells promotes lymph node metastasis and enhances glucose metabolism in tumor cells through the SNAI1/SIRT3 pathway, but PD-L1 has the opposite effect on T-cell glycolysis (42). With the application of anti-CTLA-4 or anti-PD-1 antibodies, the activity of glucose metabolism of T cells can be restored, and glycolysis in tumor cells can be suppressed by reducing the expression of mTOR and glycolysis-related genes (57). On the other hand, suppressing the expression of glycolytic genes in tumor cells can improve the effectiveness of immune checkpoint therapy (58). These findings further deepen our understanding of anti-PD-1/PD-L1 treatment and present new strategies for CAR-T cells optimization.

Furthermore, the acidified atmosphere in the TME also restricts T cells metabolism. Lactate can induce the infiltration of FOXP3-expressing regulatory T cells in the TME (59). Diclofenac, an NSAID, can block the activity of lactate transporters (monocarboxylate transporters, MCT) in tumor cells but does not affect the function of T cells and can be an adjuvant therapy for CAR-T cell therapy (60). Targeting lactate dehydrogenase-A (LDH-A) in tumor cells can reduce the level of oxidative phosphorylation in tumors and enhance the efficacy of anti-hPSMA CAR-T cells treatment (61). For other cells in the TME, inhibitory immature myeloid cells (IMCs), well-known immunosuppressive cells, enhance glycolysis in the tumor microenvironment and can perform normal inhibitory functions in low-sugar environments due to glutamine metabolism (62).

### 3.2 Amino acids

Adequate levels of extracellular arginine and tryptophan are necessary for maintaining the normal function of T cells. However, tumor cells in the TME can produce Arg1, which breaks down extracellular arginine outside of T cells, leading to a deficiency of arginine and inhibiting T cell function. Arginine is found to regulate glycolysis and mitochondrial oxidation, and exogenous addition of arginine can induce a metabolic transition from glycolysis to OXPHOS. Recently, a study indicated that T cells are sensitive to extracellular arginine due to their low expression of the arginine resynthesis pathway enzymes argininosuccinate synthetase (ASS) and ornithine transcarbamylase (OTC). A low arginine microenvironment also impairs the proliferation and function of CAR-T cells (63). Furthermore, amino acid metabolism is closely related to immunosuppressive cells in the TME. Mesenchymal stem cells (MSCs) are a typical immunosuppressive cell type that can enhance glucose metabolism under inflammation and inhibit T cell function through the JAK/STAT1 pathway by producing IDO, which decomposes tryptophan (64). Hindering the glucose metabolism of MSCs can reverse the function of the JAK/STAT1/IDO axis. Myeloid-derived suppressor cells (MDSCs) produce arginase-1 and IDO to reduce the levels of L-arginine and L-tryptophan (65). In terms of inhibiting the above enzymes, inhibition of arginase by CB-1158 blocks myeloid cell-mediated immune suppression in the tumor microenvironment (66). Furthermore, the metabolite of arginine, NO, produced by inducible NO synthase (iNOS), is a key feature of immunosuppressive bone marrow cells, which impairs T cell activation and proliferation by reversibly blocking IL-2 receptor signaling or S-nitrosylation-dependent mechanisms (59, 67, 68). Tryptophan plays a critical role in effector T cells in tumors, but it can be metabolized into the immunosuppressive metabolite L-kynurenine (Kyn) through IDO enzymes. Inhibiting IDO can increase cytotoxic immune cells, including CD8+ T cells, natural killer (NK) cells, and invariant NKT cells, while also inhibiting Th17 cells and reducing the differentiation of naïve CD4+ T cells into Tregs. The combined inhibition of IDO1, CTLA4, and CD274 (an immunosuppressive molecule best known as PD-L1) has been shown to mediate superior therapeutic effects against well-established gliomas in mice and induce the antitumor immune responses (69, 70). There are already multiple IDO1 inhibitors in clinical research, such as Indoximod, Epacadostat/INCB024360, NLG919, PF-06840003 (58, 71–73). At the ASCO 2017, it was announced that INCB024360 combined pembrolizumab treatment for advanced NSCLC can achieve ORR and disease control rates of 35% and 63%, respectively. In addition, a specific peptide vaccination directed against IDO1 can be combined with anti-PD-1 therapy to inhibit tumor cell growth. And IDO1-expressing DCs exert indeed broad and robust immunosuppressive effects *via* constitutive activation of Tregs (72). These can all be potential targets for enhancing the efficacy of CAR-T cell therapy (74).

The exact role of glutamine in T cell metabolism is not fully understood. Glutamine restriction can increase the expression of antiapoptotic genes in CD8+ T cells and the number of CD8+ T cells while still maintaining their normal function (but cannot increase the production of cytokines). Additionally, glutamine can induce the differentiation of CD8+ T cells into memory cells and enhance their response to restimulation. Interestingly, there is also evidence that glutamine influences T cell glycolysis and the maintenance of mTORC1, which implicates a glycolysis-mTORC1-glutamine interaction. Thus, while glutamine is necessary for T cell metabolism, excessive mTORC1 activation can lead to intracellular disorders. Despite the complexity of glutamine metabolism in T cells, it has been found that in the TME, glutaminolysis compensates for glucose metabolism due to glucose deficiency and increases the production of IFN-γ in T cells (75).

Some fibroblasts in the TME can impair the function of CD8+ T cells through the ERK1/2 and NF-κB pathways, enhancing the activity of L-arginase and the secretion of CXCL12, thereby increasing the expression of T cell immune receptors with Ig and ITIM domains (TIGHT) and BTLA (B and T lymphocyte attenuator) (76). Additionally, hexamine synthesis bypass is enhanced in tumor cells, which promotes survival, increases the synthesis of hyaluronic acid and collagen and inhibits the function of TILs. Although it is related to glucose metabolism, using a glutamine analogous (6-diazo-5-oxo-l-norleucine, DON) can reverse this process and increase the infiltration of CD8+ T cells, which indicates that glutamine inhibition has a positive impact on the antitumor abilities of T cells (77). GCN2 is the sensor of amino acid deficiency outside the cell, leading T cells into a blocked state, but this status is beneficial to the survival of CD8+ T cells, which may be a metabolic reprogramming caused by perception (78).

PRODH2 catalyzes the conversion of 4-hydroxyproline to 1-pyrroline-3-hydroxy-5-carboxylate (PHC). PRODH2 is typically not expressed in T cells but is expressed in the mitochondria of kidney, liver and gallbladder cells. The overexpression of PRODH2 can reprogram T cell metabolism by promoting T cell proliferation, activation and effector function, thus enhancing the antitumor effect *in vivo*. Metabolomic analysis has confirmed that the 4-hydroxyproline level of PRODH2 CD22-CAR T cells is decreased and the PHC level is increased. This work provides additional evidence for the importance of the proline metabolism pathway in T cell function (42, 57).

### 3.3 Lipids

Lipids play important roles in cell survival and proliferation, serving as signaling molecules, energy sources, and structural components of cell membranes. Dysregulation of lipid metabolism, particularly in the metabolism of fatty acids (FAs) and cholesterol, has been identified as a key metabolic rewiring event that shapes the TME. This finding highlights the importance of understanding the impact of lipid metabolism on T cells in the TME.

#### 3.3.1 Fatty acids

The impact of fatty acids (FAs) on T cells is highly dependent on species and context. In mouse melanoma models and human melanoma samples with limited glucose and oxygen, tumor-infiltrating CD8+ T cells enhance peroxisome proliferator-activated receptor (PPAR)-α signaling and catabolism of FAs through FAO as an alternative to glucose-derived pyruvate, maximizing energy production and reducing equivalents (79). Treatment with a PPARα agonist during OT-I cell *in vitro* stimulation increases the frequencies of GrmB+ and IFN-γ+ CD8+ TILs *in vivo* and markedly delays tumor progression. In human hepatocellular carcinoma (HCC) samples, TILs that have enhanced fatty acid-binding protein 5 (FABP5) expression and lipid uptake display stronger effector function and survival (80). In addition, ketone bodies (79) and short-chain fatty acid acetate (55) can serve as alternative fuels in the nutrient-limited TME as well. Supplemental acetate promotes chromatin accessibility, histone acetylation, and IFN-γ production in glucose-restricted T cells through acetyl-CoA synthetase (ACSS)-dependent production of acetyl-CoA, while little effect is seen on CD8+ T cells activation marker expression, proliferation and granzyme B expression (55). Given that acetate is an important alternative carbon source for cancer cells as well (81), more work is needed to determine the relevance of acetate utilization by T cells and tumor cells in the TME, which is essential for the generation of future therapies to promote durable T cells immunity in cancer.

On the other hand, preclinical mouse models of pancreatic ductal adenocarcinoma (PDAC) and human tumor specimens demonstrate that PDAC progression is characterized by the accumulation of specific lipids in the TME, specifically long-chain and very-long-chain fatty acids (LCFAs and VLCFAs). Excess LCFA in the TME, as well as *in vitro* treatment with palmitamide, can have a detrimental impact on T cells’ mitochondrial mass, integrity, and functionality, rendering them unable to effectively engage in the oxidative phosphorylation (OXPHOS) pathway in response to limited glycolysis (82). Mechanistically, the downregulation of VLC acyl-CoA dehydrogenase (ACADVL, enzyme that catalyzes the initial step of mitochondrial β-oxidation and targets esters of LCFAs and VLCFAs) is particularly relevant, as it impairs the ability of CD8+ T cells to metabolize excess specific LCFA lipids. Metabolic reprogramming of engineered TCR_1045_ T cells by increasing ACADVL expression can improve the persistence of effector CD8+ T cells (82), which may also be applicable to CAR-T cells against PDCA as well as other lipid-rich tumors, such as HCC and breast cancer.

In both human and murine models, intratumoral Treg cells exhibit an elevated ability to take up fatty acids, resulting in a higher lipid content. This increase in lipid uptake is due to the upregulation of CD36, a scavenger receptor responsible for the uptake of long-chain fatty acids and oxidized low-density lipoproteins. Further investigation showed that CD36-PPAR-β signaling sustains survival and function in intratumoral Treg cells by modulating mitochondrial fitness and NAD+ levels. Blocking CD36 has been shown to specifically disrupt intratumoral Treg cells, leading to reprogramming of the TME toward more immune-stimulatory conditions (83). It would be interesting to explore whether overexpressing CD36 in CAR-T cells could increase their metabolic fitness or skew the T cells toward a Treg-like phenotype.

#### 3.3.2 Cholesterol

Cholesterol is a crucial component of membrane lipids and plays a crucial role in TCR clustering, signaling, and the formation of the T-cell immunological synapse (84). Inhibiting cholesterol esterification in T cells, either through genetic ablation or pharmacological inhibition of acetyl-coenzyme A acetyltransferases (ACAT1), a key cholesterol esterification enzyme, leads to increased levels of plasma membrane cholesterol levels, leading to potentiated effector function and enhanced proliferation of CD8+ T cells in melanoma and Lewis lung carcinoma models (85). However, studies using subcutaneous melanoma, Lewis lung carcinoma, and colorectal cancer models have shown that cholesterol enriched in the TME induces CD8+ T-cell exhaustion by upregulating the expression of immune checkpoints in an ER stress-XBP1-dependent manner (86). Either inhibition of XBP1 activity or inhibition of the rate-limiting enzyme in cholesterol synthesis restores CD8+ T-cell function in the tumor microenvironment and improves their antitumor activity after adoptive transfer. Cholesterol-lowering drugs such as statins might hold promising applications. Two other studies found that TCR signaling was inhibited by cholesterol (87) or cholesterol sulfate (88) either *via* binding to the TCRβ transmembrane region to prevent switching to the active conformation or disrupting TCR multimers by replacing cholesterol bound to the TCR-CD3 complex, respectively. Given the paradoxical function of cholesterol that has been reported, the benefits of targeting specific aspects of cholesterol metabolism to improve the antitumor immune response, requires further exploration.

#### 3.3.3 PGE2 and LPA

Prostaglandin E2 (PGE2) is a lipid mediator generated from arachidonic acid by cyclooxygenase-2 (COX-2) and acts by binding to PGE2-sensitive (EP) receptors (89). COX-2 is frequently overexpressed in various cancers, leading to increased PGE2 production in the TME and promoting tumor progression and metastasis. PGE2 overproduction also inhibits CD8+ T-cell activity, promotes the development of Treg cells, and skews activated T cells toward an anti-inflammatory phenotype (89–91). Wang et al (92) reported that the activation of PGE2/EP2 and PGE2/EP4 signaling positively regulates the expression of PD-1 in infiltrating CD8+ T cells, leading to immune tolerance in the TME of lung cancer. Therefore, targeting COX2, EP receptors, or downstream PGE2 synthase 1 (PGES1) (93) may be beneficial in optimizing CAR-T cell therapy.

Lysophosphatidic acid (LPA) is generated extracellularly primarily by the activity of autotaxin (ATX) and is a bioactive lipid mediator. LPA signals through the LPA-5 receptor on CD8+ T cells to suppress the TCR-induced calcium response, cell activation, proliferation, and cytotoxicity both *in vitro* and *in vivo*. Hosts deficient in LPA5 are more resistant to lymphoma and melanoma challenge (94, 95). Targeting the ATX-LPA-LPA5 axis may be a promising strategy to reinvigorate exhausted T cells in the TME.

### 3.4 Nucleic acid

A characteristic of the TME is high levels of extracellular nucleotides, which are metabolized through the dynamic and sequential action of cell surface enzymes, also known as ectoenzymes (96). In recent years, adenosinergic signaling has been shown to be a powerful immunometabolic checkpoint in tumors (97). Extracellular adenosine triphosphate (ATP) and nicotinamide adenine dinucleotide (NAD+) are considered two crucial substrates of the ectoenzymes that produce adenosine, which have immunomodulatory functions of their own.

#### 3.4.1 ATP and adenosine

As early as the 1970s, scientists discovered that extracellular adenosine (eADO) could suppress the activity of cytotoxic T cells against EL4 lymphoma cells *in vitro* by increasing intracellular cyclic AMP levels (cAMP) (98). Hypoxia, chronic inflammation, nutrient deprivation, and clinical interventions such as radiotherapy and chemotherapy can trigger the passive release or active secretion of ATP by tumor and stromal cells, resulting in high extracellular ATP (eATP) concentrations in solid tumors (99). eATP has been shown to have a positive influence on T cell activation, function, and proliferation by binding to its receptor P2X7. It also plays a critical role in promoting the metabolic fitness and survival of memory CD8+ T cells through the activation of P2X7R (100). However, high concentrations of eATP can induce cell death, and the benefits of eATP can be counteracted by its metabolite, ADO. Two ectonucleotidases, CD73 and CD39, expressed on the surface of both tumor and immune cells, convert extracellular ATP to eADO in two separate steps. CD39 hydrolyses eATP to produce extracellular ADP and AMP; subsequently, eAMP is converted to eADO by CD73 (101). Furthermore, the adenosine receptor A2aR, which is expressed on the surface of activated T cells, can be activated by adenosine and has been shown to dampen proximal T cell receptor signaling (102), diminish IL2-dependent proliferation of T cells (103), attenuate T cell cytotoxicity and cytokine production, induce T cell anergy and expression of inhibitory receptors (104), and promote the generation of regulatory T cells (105). The effects of eATP and eADO on tumor cells and other immune cells have been reviewed in detail elsewhere (100–102).

eADO can be catabolized to inosine by adenosine deaminase (ADA) or transported into cells by the nucleoside transporters ENT1/2 or CNT1/2 (106). Recently, Wang et al (107) discovered that effector T cells can use inosine as an alternative substrate to support cell growth and function in the absence of glucose *in vitro*. In addition, some of the *in vivo* effects of inosine could be mediated by the concurrent depletion of immunosuppressive adenosine. Furthermore, hypoxanthine, derived from inosine, can be converted into uric acid, which may have additional beneficial effects on T cells (108). It is worth noting that cancer cells have diverse capacities to utilize inosine, and supplementation with inosine or PEG–ADA enhances the potency and durability of T-cell-based cancer immunotherapy in several solid tumor models, such as melanoma and neuroblastoma.

#### 3.4.2 NAD+/NADH

NAD+ and its reduced form NADH are essential coupled redox metabolites that also serve as co-substrates for diverse key enzymes (96). Stressed or damaged tissue cells release NAD+ into the extracellular space, which is then utilized by adenosine diphosphate ribosyl transferase 2.2 (ART2.2), a glycosyl-phosphatidylinositol-anchored ectoenzyme expressed on T cells, to transfer ADP-ribose to arginine residues of extracellular domains of surface proteins, a process known as ADP-ribosylation. Extracellular NAD+ (eNAD+) can suppress CTL activation *via* ADP-ribosylation of p56lck kinase (109), reduce CD8+ T cells mediated cytotoxicity *via* ADP-ribosylation of CD8-β (110), and induce T-cell apoptosis *via* ADP-ribosylation of P2X7 (111). Several other molecules have also been identified as targets of ADP-ribosylation, such as CD8, CD27, CD43, and LFA-1, accounting for reduced CD8+ T cells responses *in vitro* after incubation with NAD+ or *in vivo* after NAD+ injection of mice. It is currently unclear which of these, or other unknown targets, is primarily responsible for the observed effects. Nevertheless, it has been reported that CD4+Foxp3+ Treg cells are significantly more sensitive to NAD+ than their conventional T cell counterparts, and mice with genetic deletion of ART2.2 or P2X7 have higher numbers of Treg cells (112). As a result, further validation is needed for genetically or pharmacologically manipulating the ART2-P2X7 pathway, as well as other NAD+ signaling pathways, as the effects may vary depending on the tumor type.

NAD+ is utilized as the substrate to generate extracellular AMP (eAMP) *via* a series of enzymatic reactions catalyzed by CD38 and CD203a. The former is a NAD+ ectohydrolase that breaks down NAD+ to nicotinamide (NAM) and ADP ribose (ADPR), which can then be hydrolyzed to eADO by CD73 and interrupt T cells *via* the adenosinergic pathway (96). Thus, decreasing the NADase activity of CD38 can reverse ADO-mediated immunosuppressive effects. Furthermore, T cells with CD38 deficiency exhibited intrinsically higher NAD+ levels, increased expression of antioxidant genes and stem cell-associated genes, enhanced oxidative phosphorylation and altered mitochondrial dynamics, which greatly improved tumor control in melanoma-bearing mice (113).

### 3.5 Hypoxia

The effect of hypoxia on adaptive antitumor immunity is complicated and not straightforward (103, 114, 115). The expression of HIF-1 transcriptional complexes is upregulated to respond to CD8+ T cells activation (105). In well-oxygenated environments, the PHD enzymes catalyze posttranslational hydroxylation of HIF-1α, which is then degraded (116). Deletion or pharmacological inhibition of PHD proteins within T cells limits tumor colonization in the lungs and enhances the effectiveness of adoptive cell transfer immunotherapy (117). Furthermore, metabolite S-2-hydroxyglutarate (S-2HG) production in CD8+ T cells depends on HIF-1α when T cells respond to TCR triggering under environmental hypoxia (118). S-2HG can significantly alter CD8+ T cells differentiation through epigenetic remodeling, even augmenting T cell proliferation, long-term survival, and antitumor response after *in vivo* transfer in mouse models (20, 119).

Therefore, it is likely that hypoxia can have a positive effect on TIL effector functions in tumors. However, subsequent observations suggest otherwise. Hypoxia *via* HIF-1α can induce the expression of PD-L1 on cancer cells and tumor-infiltrating MDSCs, leading to immune escape (120, 121). Other studies have discovered that hypoxia promotes the expression of ectonucleotidases CD39 and CD73, which can transfer ATP to adenosine, are promoted on various cells in the TME (122, 123). Interestingly, Hatfield et al. has proved oxygen supplement can efficiently weaken the hypoxia-adenosine axis, enhancing the intratumoral infiltration and antitumor response of endogenously developed or adoptively transferred tumor-reactive CD8+ T cells (124).

Moreover, in addition to respiratory supplementation of oxygen, treatments that target the oxidative metabolism of tumor cells have received much attention recently, which could be another efficacious way to decrease intratumoral hypoxia and relieve the immunosuppression of the TME. The oxidative metabolism of tumor cells is a primary driver of intratumoral hypoxia (125). Inhibiting tumor oxygen consumption is associated with increased T cell infiltration and decreased T cell differentiation to a more “exhausted” phenotype (125, 126). Treatments targeting tumor cell oxidative metabolism can considerably sensitize some resistant cancers to checkpoint blockade (126, 127). However, relevant studies on adoptive cell therapy are still ongoing. Recently, Cui et al. tested CAIX-specific CAR T cells against a glioblastoma model, establishing carbonic anhydrase IX (CAIX) as a targeting hypoxia downstream signaling protein characteristically expressed in glioblastoma and found robust efficacy (128). However, it is still essential to clarify the clear role of hypoxia in metabolically regulating adaptive antitumor immunity in the future so that there are more safe and reliable strategies to overcome the obstacles of CAR-T cell therapy in treating solid tumors.

### 3.6 Mitochondria and ROS

Mitochondrial respiration is a crucial aspect of T effector cell metabolism. However, an increasing number of studies have shown that mitochondria from T effector cells can be severely impaired in the TME.

Scharping et al. demonstrated that CD8 TILs from mouse and human tumors displayed a loss of mitochondrial mass and oxidative capacity, which was correlated with decreased expression of PGC-1a over time. This inhibition of mitochondrial function subsequently hindered the proliferation and production of IFN-γ by CD8+ T cells (129). In contrast, in patients with chronic lymphocytic leukemia (CLL) who experienced complete responses, infused CAR-T cells increased mitochondrial biogenesis and function compared to nonresponders (130). Therefore, strategies for immune modulation targeting the restoration of mitochondrial biogenesis and respiratory capacity have been developed. In adoptively transferred CD8+ T cells, metabolic reprogramming through enforced PGC1a expression promotes mitochondrial biogenesis and function, leading to superior antitumor responses (129). In the CAR-T field, the inclusion of the costimulatory receptor 4-1BB in the CAR architecture also boosts mitochondrial health and biogenesis, enhancing T-cell persistence and increasing therapeutic efficacy (118, 131–134).

ROS are also indispensable for T-cell-mediated immunity. Excessive ROS production can suppress antitumor immunity. Studies have shown that persistent antigen exposure in the TME can lead to metabolic alterations characterized by impaired mitochondria with oxidative stress, resulting in the formation of terminally dysfunctional TILs called exhausted T cells (135). Accordingly, buffering mitochondrial ROS with scavengers can restore the activation of exhausted TILs in response to TCR and allow sustained proliferation and self-renewal (135, 136). However, a deficiency in mitochondrial reactive oxygen species (mROS) can prevent T cells from regulating nuclear factor of activated T-cell (NFAT) activity and increasing IL-2 expression upon stimulation (137). Therefore, ROS scavengers have the potential to enhance antitumor immunity, but their effects on different subsets of T cells should be carefully considered.

### 3.7 Others

In addition to the harmful products generated from the nutrient and energy metabolism pathways discussed above (such as lactate, kynurenine, and adenosine), other toxic metabolites produced from some tumor-specific activities in the TME can also have a negative effect on infiltrating lymphocytes.

The common occurrence of tumor cell death can produce immunomodulatory products that could be toxic and harmful. Ferroptosis, a novel form of regulated cell death characterized by iron-dependent oxidative stress and lipid peroxidation, is reported to be associated with the release of prostaglandin E2 (PGE2), which can be immunosuppressive and facilitate tumor evasion (138, 139). Additionally, areas of tumor necrosis can cause elevations in the extracellular potassium concentration, which can impair T cell receptor-driven Akt-mTOR phosphorylation and induce a functional state of nutrient deprivation (140, 141), resulting in a depression of methylation of histone marks that normally suppress stemness-associated programs. Further investigation showed that treatment of elevated potassium for antitumor T cells resulted in T cells with retained stemness *in vivo*, evidenced by self-renewal, multipotency, and persistence, thereby enabling the enhanced destruction of tumors (141). This finding notably illustrates a potential strategy for improving the efficacy of adoptive T cell therapy in the future.

Cancer cells have also been found to release a type of metabolites in a specific genetic context termed oncometabolite, which indirectly facilitates tumorigenesis through immunomodulation (142). It is reported that oncometabolite 2­HG released by malignant tumor cells of isocitrate dehydrogenase (IDH)­mutant human glioma can accumulate in the extracellular space and be taken up by human CD4+ and CD8+ T cells. This results in suppression of T cell activation and proliferation *via* perturbation of ATP-dependent TCR signaling, NFAT activity, and polyamine biosynthesis (143).

In conclusion, the generation of T cell-suppressive metabolites through distinct activities intrinsic to the TME constitutes a crucial mechanism of tumor immune evasion.

## 4 Improving CAR-T cell therapy through metabolic preconditioning

CAR-T cells have shown great potential in enhancing the immune response to cancer. *Ex vivo* expanded CAR-T usually have complex and heterogeneous phenotypes, and the majority of cells are effector memory T cells (Tem) or effector T cells (Teff), and they have a very low proportion of naïve T cells (Tn) and central memory T cells (Tcm). However, the efficacy of CAR-T cell therapy can be limited by various factors, such as antigen density loss, tumor microenvironment suppression, and intrinsic CAR-T cell exhaustion.

The characteristics of CAR-T cell exhaustion include reduced cytokine production, decreased proliferative capacity, and sustained high expression of inhibitory receptors such as PD-1, LAG-3, TIM-3, or CLTA-4 (144). To overcome these limitations, CAR-T cell therapy presents a promising opportunity to improve immune function and persistence through metabolic conditioning *in vitro*, such as the glycolysis, oxidative phosphorylation (OXPHOS), mitochondrial biogenesis, and fatty acid oxidation (FAO). Restoring CAR-T cells function could also be achieved through supplementation or pharmacological treatment after reinfusion. The strategies that enhance the metabolic fitness of CAR-T cells and optimize CAR-T cell therapy efficacy can be categorized as follows: (1) generating less differentiated Tm subsets with greater expansion and persistence; (2) strengthening the effector function of CAR-T cells; and (3) overcoming metabolic stresses imposed by the TME. While this review focuses on CAR-T cells, the principles discussed have been gleaned from adoptive cell therapy studies, including those on tumor infiltrating lymphocytes and TCR-engineered T cells.

### 4.1 Increasing persistence

Terminal differentiation during *ex vivo *expansion limits CAR-T cells intratumoral persistence and survival. So one of the challenges of CAR-T cells therapy is ensuring that the modified T cells persist in the body long enough to effectively attack cancer cells, such as altering T-cell differentiation and guiding the maintenance of long-lived memory T cells (44).

**Figure 1:**
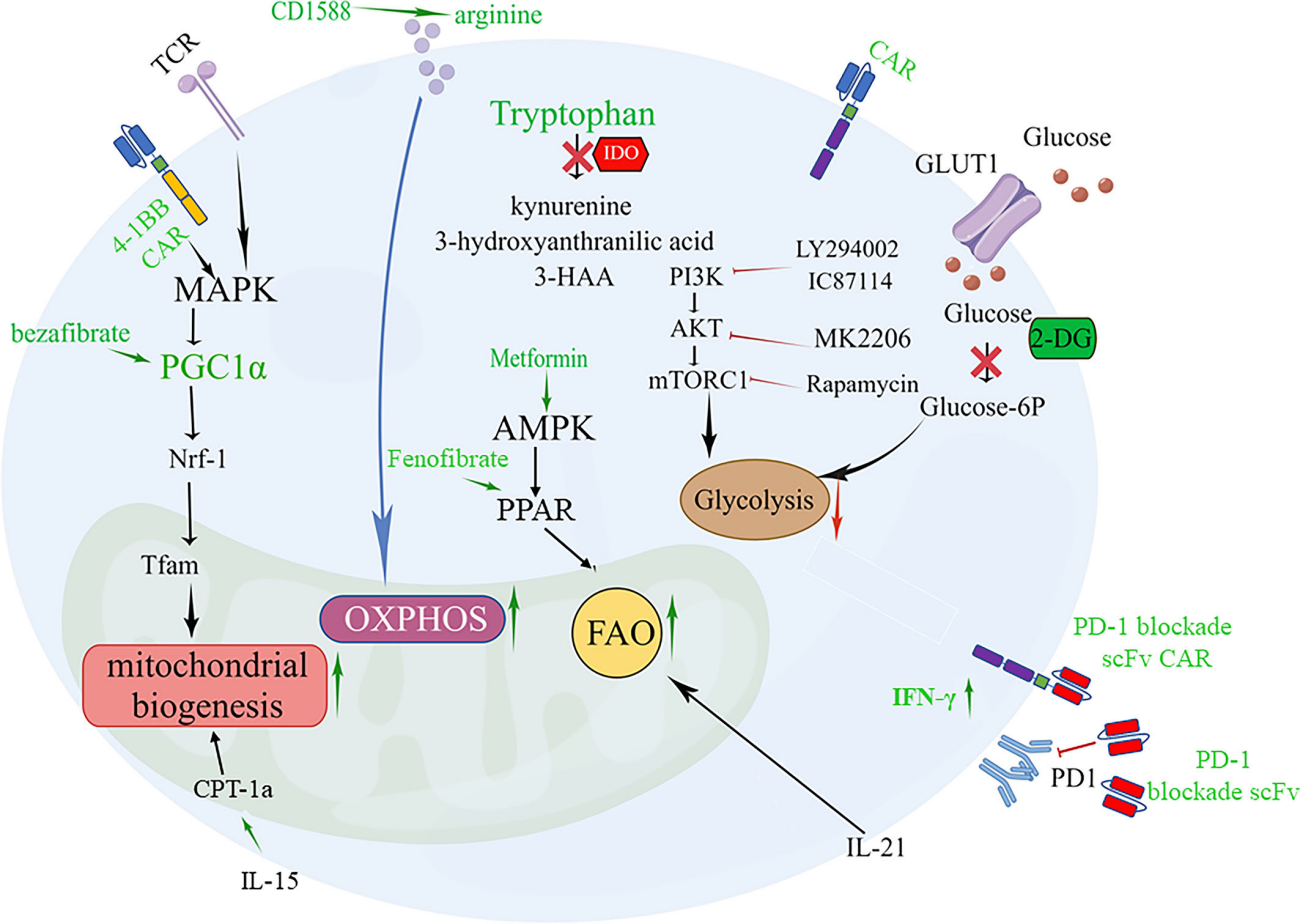
Targeting metabolic signaling that improve CAR-T cell fate and overcome exhaustion. 1.4-1BB signaling or PGC-1α activator bezafibrate upregulates PGC-1α expression through stimulation of the MAPK pathway, resulting in mitochondrial biogenesis beneficial for the differentiation of CAR-Tcm cells. PGC-1α activates NRF1, which activates Tfam involved in the transcription and replication of mtDNA. 2. Supplementation arginine enhances OXPHOS or inhibition IDO to increase tryptophan can restore the activity of CAR-T cells. 3. Promoting FAO of T cells by enhancing AMP-dependent kinases through metformin, IL-21 and PPAR agonists Fenofibrate boosts a memory-like cell differentiation trend (Tscm or Tcm). 4. Inhibition of glycolysis with 2-DG or Inhibition of PI3K/Akt/mTOR axis promotes the generation of CAR-Tcm cells. 5. Modified CAR-T cells that secrete PD-1 blockade scFv can reverse CAR-T exhaustion.

Glycolysis is a hallmark of effector/exhausted T cell metabolism, and the PI3K-Akt-mTOR pathway and GLUT1 can promote T cell glycolysis, playing a key role in regulating T cell differentiation and activity that exacerbate effector dysfunction (145–147). Moreover, CAR-T cells from patients with a poor response exhibited glycolysis gene signature transcription, and inhibition of glycolysis with 2-deoxy-d-glucose (2-DG) promoted the generation of memory CAR-T cells (**Figure 1**). Inhibiting OXPHOS can limit the self-renewal capacity of T cells and upregulate the expression of exhaustion-related genes in T cells. Therefore, enforcing metabolic reprogramming favoring mitochondrial OXPHOS over glycolysis was accompanied by T cell enhanced stemness. Blocking the PI3K-AKT-mTOR signaling pathway can protect the Tem population. PI3K inhibitors optimally maintain CAR-T cells at a low differentiation state *in vitro* without impairing their activation, significantly prolonging CAR-T cell persistence and enhancing *in vivo* antitumor activity (148). AKT inhibition had relatively little impact on glycolysis but skewed metabolism toward FAO and OXPHOS (**Figure 1**) (147).

Amino acid metabolism is involved in the antitumor efficiency of CAR-T cells. A low arginine microenvironment also impairs the proliferation of CAR-T cells. Therefore, supplement arginine for CAR-T cells, which can promote OXPHOS and inhibit glycolysis has been applied in CAR-T cell (63).Supplementation with L-arginine (65), the Arg1 antagonist CD1588 (66), or knockout of the Arg2 gene (149) and inserting the arginine resynthesis enzymes argininosuccinate synthase (ASS) or ornithine transcarbamylase (OTC) enzyme into the CAR construct that supplementing arginine for CAR-T cells can promote OXPHOS and inhibit glycolysis, thereby enhancing proliferation and activity of CAR-T cell and inducing Tcm differentiation (**Figure 1**).

Tryptophan is critical for the effector function of T cells in the tumor bed, but it can be metabolized by the enzyme IDO into immunosuppressive compounds such as kynurenine, 3-hydroxyanthranilic acid, and 3-HAA. Inhibition of IDO by administering fludarabine and cyclophosphamide can restore the activity of CD19-specific CAR-T cells against IDO-positive lymphoma *in vivo*, suggesting that IDO is a potential therapeutic target for enhancing the clinical activity of CAR-T therapy (150, 151) (**Figure 1**). The IDO1 vaccine (74), as mentioned above, can enhance the signal transmission of MHC class II and promote the differentiation of CD44^hi^CD62L^hi^ cells. Glutamine deficiency significantly reduces T cell activity and IL-2 secretion by 70-90%, resulting in impaired proliferation. Inhibiting glutamine with the glutamine antagonist 6-diazo-5-oxo-L-norleucine (DON) in the culture medium enables CAR-T cells to have enhanced mitochondrial OXPHOS and reduced glycolytic activity while preserving more Tn or Tcm subpopulations (152, 153). Furthermore, promoting fatty acid oxidation of T cells by enhancing AMP-dependent kinases through metformin (34) and promoting CPT1a expression using IL-15 (44) or PPAR α/β agonists (154) Fenofibrate boosts a memory-like cell differentiation trend (**Figure 1**).

The reserve function of mitochondria is an important factor in the differentiation of memory-like cells. For CAR-T cells, CD19-41BBζ CAR^kr^, whose intracellular lysine (K) is replaced with arginine, does not undergo ubiquitination and thus mitochondrial biosynthesis is enhanced. FAS 4-1BB IFP engineering cells, which replace the FAS tail with 41-BB, can also strengthen the function of mitochondria for long-lasting antitumor activity. CAR-T cells that express the 4-1BB signaling domain have increased PGC-1α through activation of the p38-MAPK pathway, resulting in mitochondrial fusion and biogenesis (155–158) (**Figure 1**). While CD28 costimulation promotes a more glycolytic phenotype with a faster effector response. Dual CARs with both CD28 and 4-1BB domains appear to maintain high mitochondrial metabolism while increasing glycolytic metabolism, resulting in sustained tonic TCR signaling and metabolic adaptability (159). The FDA has approved two engineered CAR-T cells with the costimulatory molecules CD28 or 4-1BB, which are reminiscent of short-lived effector T cells and long-lived memory T cells, respectively (155). Thus, engineered CAR-T cells offer a particularly promising approach to modulate T-cell metabolism.

Moreover, focusing on small metabolic molecules in cells is also important for the persistence and potency of CAR-T cells. Antioxidants (62) against ROS as well as creatine for maintaining the ATP energy pool can both promote the differentiation of Tcm cells (160). In the TME, mitochondrial pyruvate carrier (MPC) is crucial for sustaining lactate oxidation to support the antitumor function of CD8+ T cells (161). Inhibition of MPC induces metabolic flexibility that facilitates the generation of high acetyl-CoA levels through glutamine and fatty acid oxidation. This, in turn, promotes histone acetylation and chromatin accessibility on pro-memory genes. In addition, it has been shown that CAR-T cell manufacturing with an MPC inhibitor induces a memory phenotype and demonstrates that infusing MPC inhibitor-conditioned CAR-T cells resulted in efficient and long-lasting antitumor activity (155). Peroxisome proliferator-activated receptor γ coactivator 1-alpha (PGC-1α), a key regulator of mitochondrial biogenesis, inhibits the PD-1 signaling pathway, leading to CD8+ T cell exhaustion (162). In terms of therapy, the PGC-1α activator bezafibrate is beneficial for the differentiation of CD8+ Tcm cells, while PGC-1α-overexpressing exhausted T cells exhibit mitochondrial biogenesis and greater antitumor immune capacity (163) (**Figure 1**). Lentivirus carrying engineered PGC-1α in CAR-T cells can improve their cellular therapy against solid tumors (164).

Furthermore, by focusing on epigenetics, we can develop strategies to better transform T cells. The methylation and acetylation of histones has received increasing attention. The histone deacetylase 6-specific inhibitor (HDAC6i) tubastatin A, combined with acetate (50), potassium supplement (141) and GSK-3β inhibitor (45), regulates epigenetic modification and has proven practical in ACT treatment. Indeed, intervening in epigenetic processes during CAR-T cells manufacturing with small molecules or metabolites is an emerging strategy to either prevent the loss of CAR expression and exhaustion or to induce a memory phenotype (165). The bromodomain and extra terminal domain (BET) family of chromatin adapter play a role in down-regulation of CAR expression. The blocking of BET protein can improve the exhaustion phenotypes of CAR-T cells. JQ1, a BET inhibitor, reduces the level of TET2 methylcytosine dioxygenase and rescues CAR expression (166). It was reported that overexpression of the TET2 catalytic domain suppressed CAR-T cells proliferation, upregulated PD-1 and suppressed effector cytokine production (167). So BET inhibitor provides a mechanism for connecting TET2 and T cells dysfunction and change the CAR-T metabolism by ameliorating suppression of mitochondrial respiration and glycolysis in dysfunctional CD8+ CAR-T cells from patients with CLL (166). Therefore, regulating the BET epigenetic readers can improve the efficacy of cell-based immunotherapy.

Besides, the composition of cytokines in the culture medium also affects the efficacy of CAR-T cell products. IL-21 regulates the induction of Tcm cells, enhancing anti-tumor efficacy in a FAO dependent manner (168) (**Figure 1**). IL-15 are associated with the maintenance of Tm cells. L-15 enhances SRC and oxidative metabolic rate by increasing mitochondrial biogenesis and CPT1a expression (169)(**Figure 1**). The strategy of combining these cytokine components in cell culture medium shows the potential to increase Tscm or Tcm populations and improve anti-tumor efficacy.

### 4.2 Enhancing effector function

Metabolic reprogramming of tumor cells creates a hostile microenvironment, leading to dysfunction and exhaustion of tumor-infiltrating lymphocytes (TILs). However, alternative nutritional supplements may overcome this phenomenon and enable CAR-T cells to function effectively for the clearance of tumors *in vivo*. For example, treating T cells with acetate as an alternative to glucose *ex vivo* can help hyporesponsive T cells regain the ability to generate adequate IFN-γ and function effectively (170).

In the solid tumor niche, particularly in areas with increased inflammatory activity, T cells often encounter an environment with abundant reactive oxygen species (ROS), which substantially impair T cell activation, proliferation and effector differentiation. To overcome this threat, researchers have developed several genetic methods, such as targeting PAC1 (a downstream effector of extracellular ROS in T cells), co-expressing catalase along with a tumor-specific CAR, or scavenging ROS by NAC (N-acetylcysteine) treatment to increase CAR-T cell antioxidative capacity and improve bystander protection of non-transfected immune effector cells (47, 171, 172). Additionally, upregulation of the HIF-1α pathway has been shown to improve the function of T effector cells. By fusing an oxygen-sensitive subdomain of HIF1α to a CAR scaffold, CAR-T cells that are locally responsive to a hypoxic environment can be generated, which could be a promising therapeutic approach for restoring T cell function during cancer (173). ATP synthase inhibitory factor 1 (ATPIF1) is a mitochondrial protein that inhibits F_1_F_o_-ATP synthase (174), which lead to decreased glycolysis and increased oxidative phosphorylation (OXPHOS). ATPIF1 overexpression in T cells can increase the expression of IFN-γ and granzyme B, the subset of central memory T cells in CAR-T cells, and the survival rate of NALM-6 tumor-bearing mice (175).

### 4.3 Eliminating negative regulation

Toxic and immunosuppressive metabolites generated in the microenvironment can also lead to immune escape. Genetic modifications have been employed to alleviate lipid toxicity. Deletion of LPA5104 or XBP-194 in CD8+ T cells has proven effective in enhancing antitumor responses mediated by adoptively transferred T cells in melanoma-bearing mice by inhibiting the excessive generation of toxic LPA and cholesterol, respectively (86, 95). Reinforcing ACADVL expression also improves the function of effector CD8+ T cells in PDA models by inhibiting LCFA accumulation, which gives hope for future applications of CAR-T therapy in lipid-rich solid tumors (82). Moreover, strategies targeting harmful adenosine production in the TME have also been successfully exploited in several ACT or CAR-T models, including selectively targeting adenosine pathway-relevant molecules (165, 176–178) or breathing oxygen (60%) before or during adoptive T-cell treatment (124). Of note, molecular targeting of the adenosine receptor A2aR may be more effective than pharmacological inhibition in enhancing CAR-T cell activity. Stable expression of an shRNA sequence ensures constant downregulation of A2aR after T cell infiltration into the TME, whereas the concentrations of a systemically administered inhibitor may not reach sufficient levels in tumor tissue. However, evidence for improving adoptive T cell therapy efficacy by prohibiting LDH accumulation within the TME is still lacking and requires further exploration. In addition, to enhance CAR-T cell antitumor activity, it is reasonable to combine CAR-T cell therapy with PD-1 blockade, which can alleviate CAR-T cell exhaustion. This can be achieved by using PD-1 blockade antibodies or modified CAR-T cells that secrete PD-1 blockade single-chain variable fragments (scFv) in PD-L1+ hematological and solid tumor clinically relevant syngeneic and xenograft mouse models (179), which can increase glucose concentration in TME to favor T cell glycolysis and the production of IFN-γ (**Figure 1**). Therefore, it is feasible to extend this immune-regulatory CAR-T cell approach by utilizing scFvs targeting other molecules, such as LAG-3, TIM-3, or CLTA-4, and combination strategies.

Overall, alternative nutritional supplements and genetic methods hold great potential in improving CAR-T cell function and enhancing their efficacy against tumors, particularly in the hostile TME.

## 5 Conclusion and future perspectives

Changes in metabolism play a crucial role in shaping the extent and nature of T cell differentiation and function, with a metabolically hostile TME often leading to infiltrated T cell dysfunction, which seems to be the most prominent roadblock in CAR-T cell therapy against solid tumors. Understanding this metabolic control promises to improve the ability to modulate CAR-T cell efficacy and persistence, resulting in better and broader clinical benefits in patients with diverse cancers. However, our current knowledge of immune cell metabolism mostly comes from studies *in vitro* and animal models, and its applicability to humans needs further validation. Additionally, several obstacles, such as metabolic redundancy, metabolic heterogeneity, and paradoxical functions of metabolites and enzymes, must be addressed.

Fortunately, advancements in multi-omic approaches and single-cell profiling technologies have given researchers the power and confidence to address these challenges. New technologies such as organoid technology have opened new avenues for exploring the role of metabolism in the immune system and tumors. The next generation of approaches may include direct single-cell metabolite profiling, as well as the development of robust methods to isolate individual cells without obvious metabolic perturbations (180, 181). These technologies would not only enable us to identify novel targets but also develop personalized strategies for patient-tailored CAR-T cell therapy. For instance, stable isotope resolved metabolomics (SIRM) has been applied to assess the metabolic activities of thin tumor tissue slices, together with RNA-seq and metabolomics-edited transcriptomic analysis (META), enabling us to develop patient-tailored strategies to ensure the robustness and reproducibility of personalized CAR-T cell (182). In addition to improving efficacy and persistence, metabolic interventions may also help attenuate CAR-T cell treatment-related toxicities (161). The principle of ‘on/off switches’ predicated on the administration of small-molecule agents and suicide gene systems (183) can be theoretically extended by engaging metabolites or metabolic enzymes to control T cell activity spatially and temporally. Finally, beyond CAR-T cell therapy, these potential metabolic modulations can also be applied to other immunotherapies, including checkpoint blockade and cancer vaccines.

## Author contributions

WL: Writing – review & editing, Funding acquisition. XP: Writing – editing & original draft. LC, HC, YS, MS, and SM: Writing – original draft. YP: Writing – review. JW: Supervision. FL: Writing – review & editing, Conceptualization, Supervision. JL: Conceptualization, Supervision, Funding acquisition. NL: Supervision. All authors contributed to the article and approved the submitted version.
